# Larger Valve Size is Associated with Permanent Pacemaker Implantation in Edwards SAPIEN 3™ Transcatheter Aortic Valves

**DOI:** 10.7759/cureus.4370

**Published:** 2019-04-02

**Authors:** Mansoor Ahmad, Jay N Patel, Minchul Kim, Timir Baman, Marco Barzallo, Sudhir Mungee

**Affiliations:** 1 Internal Medicine, University of Illinois College of Medicine at Peoria, Peoria, USA; 2 Cardiology, University of Illinois College of Medicine at Peoria, Peoria, USA

**Keywords:** permanent pacemaker, transcatheter aortic valve replacement (tavr), edwards sapien 3 (esv3) valves

## Abstract

Background: Transcatheter aortic valve replacement (TAVR) can be complicated with a high-degree atrioventricular (AV) block requiring a permanent pacemaker (PPM) in 5% - 25% of patients. TAVR valve size is a modifiable risk factor for permanent pacemaker implantation (PPI) after TAVR. We studied the association of valve size in PPI with Edwards SAPIEN 3™ (ESV3) (Edwards Lifesciences LLC, Irvine, CA, USA) TAVR valves.

Method: This is a single-center retrospective cohort analysis of 449 patients undergoing TAVR from December 2012 to April 2018. We excluded patients with TAVR valve types other than the ESV3 (113 patients) and those with prior PPM or implantable cardioverter defibrillator (37 patients). Patients with an aborted procedure and surgical conversion were also excluded (16). Data of 14 patients with missing values for different clinical variables were excluded as well. The final sample size was 269. The primary outcome was PPI. Statistical analysis was done using Chi-square, T-test, and multivariate regression analysis. Multivariate analysis was done for comparison between different clinical variables.

Results: Of the 269 patients (50.6% males; mean age of 79.5 ± 8.7 years; mean Society of Thoracic Surgeons (STS) score: 6.2), PPI was seen in 17 patients (6.3%). Time to PPI was 1.3 days. PPI was significantly high in patients with prior conduction defects (p = 0.016). There was a positive relationship between PPI and valve size for ESV3. PPI was seen in eight patients (47%) with 29 mm valves, six patients (35%) with 26 mm valves, three patients (17%) with 23 mm valves, and none with 20 mm valves. When taken as a reference, 23 mm valves had a significantly lower PPI compared with 29 mm valves (eight versus three; p = 0.04).

Conclusion: Larger valve size is a possible risk factor for a high-degree AV in patients receiving ESV3.

## Introduction

Transcatheter aortic valve replacement (TAVR) is an approved therapy for severe aortic stenosis in patients who are deemed at intermediate or high-risk for surgical aortic valve replacement (SAVR) [1]. TAVR has known to improve one-year survival by at least 20% compared with conservative management [1]. Over the years, the use of TAVR has increased in comparatively healthier patients. It is currently under trial as an alternative for low-risk patients with aortic stenosis [2].

A high-degree atrioventricular (AV) block requiring a permanent pacemaker (PPM) has been a known complication of TAVR, occurring in 5% - 25% patients depending on different factors [3-4]. The proposed mechanism is the compression of the conduction bundle by the TAVR valve. Multiple risk factors are identified, leading to a permanent pacemaker implantation (PPI) after TAVR, including prior conduction abnormalities [5], valve type, valve size [6-7], depth of valve implantation [5, 8], prosthesis to left ventricle outflow tract diameter ratio, and the left ventricular end-diastolic diameter [4, 9].

When compared with SAVR, TAVR has shown better three-year clinical outcomes, including all-cause mortality, the incidence of stroke, and aortic valve hemodynamics [10]. However, these benefits are offset by a much higher PPI rate of 5% - 25%, as SAVR has a PPI of 1% per year [11]. In addition, if a patient requires a pacemaker after TAVR, it not only lengthens the stay in the intensive care unit and the total hospital stay, but it has also been shown to increase heart failure readmissions and mortality at one year after TAVR [12]. Thus, modifiable risk factors for PPI in TAVR are of special interest. Appropriate valve size selection is one of these factors. 

Paravalvular leak after TAVR is a significant complication that also increases mortality after TAVR [13]. The use of a comparatively larger sized TAVR valve decreases paravalvular leaks [13]. This valve oversizing, however, increases the PPI rate after TAVR. Among self-expanding TAVR valves, oversizing is an independent risk factor for PPI [7]. Studies have shown mixed results for valve oversizing, leading to PPI in balloon expandable valves [6, 14]. Although studies comparing PPI in recent generations of balloon expandable valves are emerging [15], data looking at the association of valve size and PPI in Edwards SAPIEN 3™ (ESV3) valves (Edwards Lifesciences LLC, Irvine, CA, USA) are limited. This study will focus on the association between valve size and PPI in ESV3 TAVR valves.

## Materials and methods

Patient population and study design

We did a retrospective chart review of 449 patients who received TAVR at OSF Saint Francis Medical Center between December 2012 and April 2018. Institutional Review Board approval was obtained from the office of Human Research at the University of Illinois Chicago at Peoria, IL. Considering the retrospective nature of this study, a consent waiver was approved. All patients undergoing TAVR were deemed at intermediate or high-risk for SAVR by the local cardiothoracic surgery team based on the Society of Thoracic Surgeons (STS) score.

The initial sample size was 449. Patients who received TAVR valves, other than the ESV3, were excluded (Edwards SAPIEN - 44 patients and Edwards SAPIEN XT - 69 patients). Of the remaining 336 patients, we excluded 37 patients with prior PPM or an implantable cardioverter defibrillator. Patients with the aborted procedure or those requiring cardiopulmonary bypass or surgical conversion (16 patients) were also excluded, as these patients either did not receive the TAVR valve or valve area was surgically manipulated. Data of 14 patients with missing values for different clinical variables were excluded as well. The final sample size was 269.

Clinical, electrocardiographic, and echocardiographic data

Clinical data were extracted retrospectively, and every patient had a baseline electrocardiogram (EKG) and echocardiogram done before TAVR. Clinical variables studied included age, gender, body mass index (BMI), STS score, history of hypertension, diabetes, prior myocardial infarction, heart failure with different New York Heart Association functional classes (NYHA Class), atrial fibrillation or flutter, smoking, chronic lung disease, use of home oxygen, and renal disease requiring dialysis. In addition, we looked at the pre-procedure hemoglobin and creatinine levels.

On EKG, conduction defects were defined as the presence of a right bundle branch block (RBBB), a left hemi-fascicular block, bi-fascicular block, first-degree AV block, or second-degree AV block Type I (Mobitz-Type I). Echocardiographic variables included left ventricular internal diameter measured at systole and diastole (LVIDs/LVIDd) and ventricular septal wall thickness.

Device description

This study is solely based on Edwards SAPIEN 3 TAVR valves.

Criteria for pacemaker implantation

The major indication for PPI was a high-degree AV block which did not resolve following TAVR. Temporary pacing was used when necessary. The timing of PPI was extracted from the chart review.

Outcome comparison

The primary outcome was the implantation of a PPM. 

Statistical analysis

Patients were divided into two groups based on PPI status. Baseline characteristics and clinical data were compared among groups. Continuous data were represented as mean ± standard deviation (SD) and categorical data as proportions. A T-test was used to compare continuous variables and the Chi-square test for categorical variables.

Adjusted statistical analyses were conducted for the clinical outcomes. A multivariable analysis was performed to evaluate clinical predictors. Only 255 patients were used for multivariate regression analysis due to collinearity. For logistic analysis of the PPM outcome, the following variables were omitted due to collinearity: valve size (20 mm valves only), dialysis, and year (Table 1).

**Table 1:** Multivariate Analysis for Pacemaker Implantation in Edwards SAPIEN 3 Valve Valve size of 20 mm (14 observations) omitted due to no observation in the PPM group (Outcome: PPM, n=255). A fib: atrial fibrillation; BMI: body mass index; Cr: creatinine; Hb: hemoglobin; HF: heart failure; LVIDd: left ventricular internal diameter diastolic; LVIDs: left ventricular internal diameter systolic; MI: myocardial infarction; NYHA: New York Heart Association, O_2_: oxygen; PPM: permanent pacemaker; STS: Society of Thoracic Surgeons

Covariates	Odds Ratio	P value	95% Confidence Interval	
Age	1.03	0.623	0.92	1.15
Male	0.09	0.088	0.01	1.44
Smoker	22.56	0.068	0.80	640.04
STS score	1.08	0.460	0.88	1.33
BMI (Ref: Normal)				
Underweight	2.01	0.603	0.14	27.92
Overweight	12.77	0.024	1.39	117.25
Obese	15.02	0.036	1.19	189.92
Hb pre-procedure	1.73	0.025	1.07	2.79
Cr. pre-procedure	0.68	0.552	0.19	2.40
LVIDs	0.41	0.311	0.07	2.30
LVIDd	3.12	0.246	0.46	21.22
Septal wall ≥ 1.1 (Ref: < 1.1)	0.68	0.728	0.08	5.82
Valve size (Ref: 23 mm)				
26 mm	8.34	0.078	0.79	88.32
29 mm	20.21	0.046	1.06	385.36
Moderate anesthesia	0.64	0.642	0.10	4.26
Prior NYHA III-IV	0.57	0.585	0.07	4.32
Chronic lung disease				
Mild	1.48	0.675	0.24	9.31
Moderate	1.22	0.853	0.15	10.07
Severe	0.01	0.096	9.3E-05	2.12
Diabetes	0.72	0.665	0.16	3.21
Home O_2_	1.11	0.944	0.06	22.52
Immunosuppression	1.37	0.821	0.09	20.33
Prior MI	1.18	0.836	0.25	5.65
Prior HF	2.33	0.448	0.26	20.75
Hypertension	0.19	0.168	0.02	2.00
A fib/flutter	2.74	0.160	0.67	11.13
Conduction Defect	14.40	0.009	1.97	105.13

All calculations were performed using Stata software, v12 (StataCorp LLC, College Station, Texas, USA), and a p-value of less than 0.05 was considered statistically significant.

## Results

Baseline demographics

Of the total 269 patients, PPI was seen in 17 patients (6.3%). The mean patient age was 79.5 years (SD: 8.7 years) with 50.6% being male (Table 2). The majority had the procedure done through femoral access (86%). The type of access did not change the PPI rate in our study (p-value = 0.915). 

**Table 2:** Baseline Characteristics Chi-square test for categorical variables and t-test for continuous variables # of samples (proportion % by column) Mean (SD) A fib: atrial fibrillation; Cr: creatinine; Hb: hemoglobin; HF: heart failure; LVIDd: left ventricular internal diameter diastolic; LVIDs: left ventricular internal diameter systolic; MI: myocardial infarction; NYHA: New York Heart Association, O2: oxygen; PPM: permanent pacemaker; STS: Society of Thoracic Surgeons

Variables	All samples (N = 269)	PPM (N = 17)	No PPM (N = 252)	P-value*
Age	79.5 (8.7)	79.5 (8.7)	80.6 (8.7)	0.591
Male	136 (50.6%)	11 (64.7%)	125 (49.6%)	0.228
Smoker	13 (4.8%)	2 (11.7%)	11 (4.4%)	0.169
Hypertension	246 (91.4%)	14 (82.3%)	232 (92.1%)	0.166
Diabetes	120 (44.6%)	7 (41.2%)	113 (44.8%)	0.769
Home O_2_	11 (4.1%)	1 (5.9%)	10 (3.9%)	0.700
Immunosuppression	20 (7.4%)	1 (5.9%)	19 (7.5%)	0.801
Prior MI	85 (31.6%)	6 (35.3%)	79 (31.4%)	0.735
Prior HF	39 (14.5%)	3 (17.6%)	36 (14.3%)	0.703
A fib/flutter	99 (36.8%)	8 (47.1%)	91 (36.1%)	0.365
Conduction Defect	130 (48.3%)	15 (88.2%)	115 (45.6%)	0.001
Conscious Sedation	178 (66.2%)	11 (64.7%)	167 (66.3%)	0.895
Body Mass Index	30.3 (7.7)	32.9 (6.4)	30.1 (7.7)	0.037
Underweight (< 25)	67 (24.9%)	2 (11.8%)	65 (25.8%)	
Normal (25 ~ < 30)	87 (32.3%)	2 (11.8%)	85 (33.7%)	
Overweight (30 ~ < 35)	60 (22.3%)	7 (41.2%)	53 (21.0%)	
Obesity ( ≥ 35)	55 (20.5%)	6 (35.3%)	49 (19.4%)	
Prior-NYHA 4 category				0.966
I	2 (0.7%)	0 (0.0%)	2 (0.8%)	
II	28 (10.4%)	2 (11.7%)	26 (10.3%)	
III	121 (44.9%)	7 (41.2%)	114 (45.2%)	
IV	118 (43.8%)	8 (47.1%)	110 (43.6%)	
Prior-NYHA 2 category				0.934
I-II	30 (11.1%)	2 (11.8%)	28 (11.1%)	
III-IV	239 (88.8%)	15 (88.2%)	224 (88.9%)	
Chronic lung disease				0.951
None	154 (57.3%)	9 (52.9%)	145 (57.5%)	
Mild	53 (19.7%)	4 (23.5%)	49 (19.4%)	
Moderate	41 (15.2%)	3 (17.6%)	38 (15.1%)	
Severe	21 (7.8%)	1 (5.9%)	20 (7.9%)	
STS score	6.2 (5.9)	6.7 (5.9)	6.2 (4.9)	0.687
Hb pre-procedure	12.1 (1.7)	12.9 (1.9)	12.0 (1.6)	0.021
Cr pre-procedure	1.3 (0.9)	1.2 (0.7)	1.3 (0.9)	0.689
LVIDs	3.2 (0.8)	3.1 (0.8)	3.2 (0.8)	0.669
LVIDd	4.6 (0.7)	4.8 (0.9)	4.6 (0.7)	0.371
Septal wall				0.414
< 1.1	52 (19.3%)	2 (11.8%)	50 (19.8%)	
≥ 1.1	217 (80.7%)	15 (88.2%)	202 (80.2%)	

Clinical variables and PPI

PPI was significantly higher in patients with prior conduction defects (88.2% vs. 11.8%: p = 0.001). The average BMI was 30.3 (SD: 7.1). A larger number of PPIs were seen in patients with a BMI ≥ 30 compared with those who had a BMI < 30 (13 vs 4; p = 0.037). Pre-procedure hemoglobin was slightly higher in the PPM group, which was statistically significant (12.9 vs. 12.0; p = 0.21). No other clinical variable was statistically significant.

Valve size and PPI

**Figure 1:**
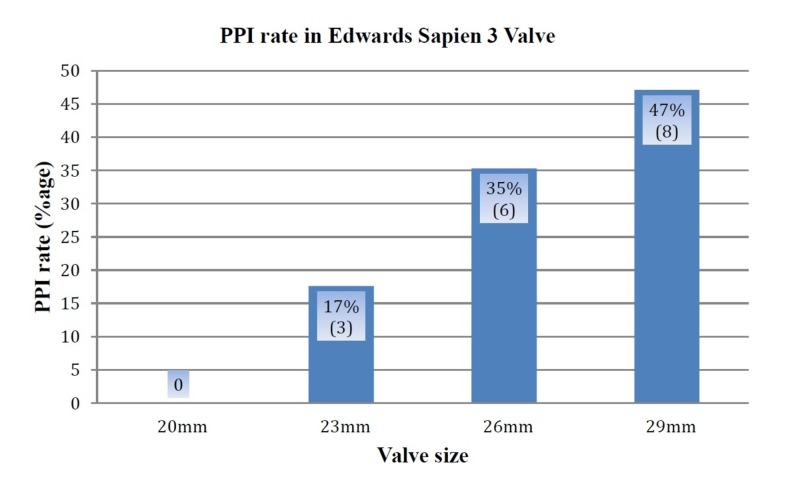
New Permanent Pacemaker Implantation (PPI) in Edwards SAPIEN 3 TAVR Valves Percentage of total pacemakers (# of pacemakers) TAVR: transcatheter aortic valve replacement

There was a positive relationship between increasing valve size and PPI (Figure 1, Table 3). The multivariate analysis for the Edwards SAPIEN 3 valves, which included 255 patients (Table 1), showed that the PPI rate was statistically significant (p = 0.04) for patients with a 29 mm valve when compared with 23 mm valves (47% vs 17.6%, respectively). Although there was a clinically stronger relationship between 29 mm and 20 mm valves (47% vs 0%, respectively), the observations with 20 mm valve size were dropped from the statistical analysis as no PPM implantation was seen for 20 mm valves. Although the PPI rate was higher in the 26 mm valve compared with 23 mm (35% vs 17%, respectively), it did not reach statistical significance (p = 0.06).

**Table 3:** Pacemaker Implantation for Valve Size in Edwards SAPIEN 3 Valve (ESV3) # of the sample (proportion for the column) PPM: permanent pacemaker

ESV3 Valve Size (mm)	Total	New PPM	No PPM
20	14 (5.2%)	0 (0.0%)	14 (5.6%)
23	93 (34.6%)	3 (17.6%)	90 (35.7%)
26	99 (36.8%)	6 (35.3%)	93 (36.9%)
29	63 (23.4%)	8 (47.1%)	55 (21.8%)

Predictors of PPI

Analyzing the baseline clinical variables (Table 2), the PPM group had a significantly higher percentage of patients with prior conduction defects (88.2% vs 11.8%: p = 0.001). On multivariate regression (Table 1), the odds of receiving a PPM were 14.4 times higher if the patient had a prior history of conduction defects (odds ratio (OR): 14.4, CI: 1.97 - 105.1, p = 0.009). A higher hemoglobin value prior to TAVR increased the odds of receiving a PPM (OR: 1.73, CI: 1.07 - 2.79, p = 0.025). We found that patients who were overweight (BMI > 30) and obese (BMI ≥ 35) had a significantly higher chance of receiving a PPM compared with those with normal BMI (OR: 12.77 and 15.02, respectively).

## Discussion

Pacemaker implantation adds a considerable cost and morbidity to TAVR admissions. It has also been shown to increase mortality in TAVR patients by about 12% at one year [9, 12]. This study looks at the association between TAVR valve size and PPI exclusively using the Edwards SAPIEN 3 valves. 

Data show that the use of a larger TAVR valve is associated with higher PPI in mechanical self-expanding valves. A study by Schroeter et al. found that valve oversizing is an independent risk factor for new PPI with mechanical self-expanding valves [7]. However, for balloon expandable TAVR valves, data are not so robust when it comes to valve sizes as a risk factor for PPI. A study by Binder et al. with balloon expandable valves involving Edwards SAPIEN XT valves shows that annular area oversizing was not associated with new conduction disturbances and PPI [8]. However, a recent study by Sheth et al. depicts that using a smaller ESV3 with selective overfilling of the deployment balloon to avoid oversizing (> 12% - 15% for nominal valve size) is associated with a low rate of PPI [6]. Thus, being a modifiable risk factor, valve size is a topic of discussion in TAVR patients. 

Foreshortening of the valve, which is a difference in valve height on balloon expansion at deployment, is intrinsically smaller for smaller TAVR valve sizes compared with larger valves. For example, a 23 mm ESV3 valve shortens from a height of 24.5 mm to 18 mm after deployment, with foreshortening of 6.5 mm, this number is 8.5 mm for 29 mm ESV3 (valve height from 31 mm to 22.5 mm). Thus, the height of the TAVR valve after deployment will be longer in a larger valve (18 mm for 23 mm valve vs. 22.5 mm for 29 mm valve). This could be one of the reasons for compression of the conduction bundle in larger valves as they will have a tendency to finish deeper in the ventricle after deployment.

In addition, ESV3 has an outer skirt to prevent a paravalvular leak which was not present in its predecessor, the Edwards SAPIEN XT; this could be an additional factor compressing the conduction bundle after TAVR. Data do show a higher PPI in ESV3 compared with SAPIEN XT valves [14].

There is very limited literature that reviews PPI with a breakdown for valve size, especially for ESV3. The relationship between PPI and valve size in our study is positive through the whole range of valve sizes (20 mm to 29 mm). Although our findings are statistically significant for 29 mm ESV3 when compared with the 23 mm valve, it does reach near-significance when the 26 mm valve group is compared with the 23 mm valve group (p = 0.07). 

Although patients with 20 mm valve size were excluded from statistical analysis because there was no pacemaker placement in this group, PPI with 29 mm valves (8 PPMs) and 26 mm valves (6 PPMs) is considerably higher than the 20 mm valves (0). Thus, these findings are clinically relevant. It is also noteworthy that the 20 mm valve, being the smallest size, is a very infrequently used valve size and we have only 14 patients receiving this valve over a five-year period.

In addition to valve size, this study looked at different clinical predictors and strongly validates conduction disturbances as a predictor of PPI post-TAVR. It was statistically significant both with Chi-square and with linear regression analysis. Prior studies show that preexisting conduction disturbances are an independent risk factor for a complete AV block after TAVR [16].

Multivariate regression shows that the odds of PPI are higher with a higher BMI and hemoglobin level at the time of TAVR. Data looking at this association are not so robust, and this could be the topic of interest for the future.

Strengths and limitations of this study

This study is exclusive to ESV3, which is one of the most common TAVR valves being used recently, and data comparing PPI in ESV3 are very limited. 

Being a retrospective review, this study has its inherent limitations. Although our cohort size is decent compared with many single-center experiences, we cannot apply the results to a more diverse patient population. Being exclusive to ESV3, the results of this study cannot be generalized to other TAVR valve types. The lack of any PPM in the 20 mm valve size required removal of this group from the multivariate regression, and thus, we cannot run a statistical analysis on this group. This study did not compare valve implantation depth between the two groups, which is an important factor in PPI after TAVR.

## Conclusions

A larger TAVR valve size is associated with a higher odds of pacemaker implantation in Edwards SAPIEN 3 valves. Improvement in device design and modifications in deployment techniques would help refine this outcome. 
